# Current Triple-Negative Breast Cancer Subtypes: Dissecting the Most Aggressive Form of Breast Cancer

**DOI:** 10.3389/fonc.2021.681476

**Published:** 2021-06-16

**Authors:** Miquel Ensenyat-Mendez, Pere Llinàs-Arias, Javier I. J. Orozco, Sandra Íñiguez-Muñoz, Matthew P. Salomon, Borja Sesé, Maggie L. DiNome, Diego M. Marzese

**Affiliations:** ^1^ Cancer Epigenetics Laboratory at the Cancer Cell Biology Group, Institut d’Investigació Sanitària Illes Balears (IdISBa), Palma, Spain; ^2^ Saint John’s Cancer Institute, Providence Saint John’s Health Center, Santa Monica, CA, United States; ^3^ Keck School of Medicine, University of Southern California, Los Angeles, CA, United States; ^4^ Department of Surgery, David Geffen School of Medicine, University California Los Angeles (UCLA), Los Angeles, CA, United States

**Keywords:** triple-negative breast cancer, TNBC, molecular subtype of breast cancer, epigenetics, clustering, artificial intelligence-AI, classification, precision medicine

## Abstract

Triple-negative breast cancer (TNBC) is a highly heterogeneous disease defined by the absence of estrogen receptor (ER) and progesterone receptor (PR) expression, and human epidermal growth factor receptor 2 (HER2) overexpression that lacks targeted treatments, leading to dismal clinical outcomes. Thus, better stratification systems that reflect intrinsic and clinically useful differences between TNBC tumors will sharpen the treatment approaches and improve clinical outcomes. The lack of a rational classification system for TNBC also impacts current and emerging therapeutic alternatives. In the past years, several new methodologies to stratify TNBC have arisen thanks to the implementation of microarray technology, high-throughput sequencing, and bioinformatic methods, exponentially increasing the amount of genomic, epigenomic, transcriptomic, and proteomic information available. Thus, new TNBC subtypes are being characterized with the promise to advance the treatment of this challenging disease. However, the diverse nature of the molecular data, the poor integration between the various methods, and the lack of cost-effective methods for systematic classification have hampered the widespread implementation of these promising developments. However, the advent of artificial intelligence applied to translational oncology promises to bring light into definitive TNBC subtypes. This review provides a comprehensive summary of the available classification strategies. It includes evaluating the overlap between the molecular, immunohistochemical, and clinical characteristics between these approaches and a perspective about the increasing applications of artificial intelligence to identify definitive and clinically relevant TNBC subtypes.

## Introduction

Breast cancer (BC) is the most prevalent cancer in women, with a steadily increasing number of cases diagnosed every year (1). Traditionally, BC is classified and treated based on the status of estrogen receptor (ER), progesterone receptor (PR), and human epidermal growth factor receptor 2 (HER2) expression. The presence of these markers has allowed the development of targeted and efficient therapies. Tumors without expression of ER, PR, and overexpression of HER2, collectively known as triple-negative breast cancer (TNBC), lack targeted therapies, leaving chemotherapy as the only systemic therapeutic alternative (2). TNBC exhibits a higher proliferation rate, higher incidence of metastases to the brain, liver, and lungs (3), and more often affects younger patients (4) than the other BC subtypes. This aggressiveness, added to the absence of targeted therapies, maintains TNBC as an unmet clinical challenge.

One of the main strategies employed to improve precision oncology involves a better understanding and rational classification of malignancies. During the last 20 years, researchers have characterized BC tumors and classified them into intrinsic molecular subtypes (5, 6). In these pivotal studies, the basal-like subtype was associated with the absence of ER and HER2 expression, and a higher expression of basal cytokeratin (CK 5/6). This classification, currently performed by the PAM50 test (7), is well accepted in the clinical setting. The TNBC and the PAM50 Basal-like subtype have frequently been considered synonymous; however, this is not always the case. Although nearly 85% of PAM50 basal-like tumors have a TNBC phenotype, the remaining 15% exhibit other express ER, PR, or HER2 markers (8–10).

Despite TNBC being grouped as a single disease, clinical, histological, and molecular profiling highlight its intrinsic heterogeneity. Nevertheless, from a clinical perspective and based purely on the absence of ER, PR, and HER2 positivity, patients with TNBC are uniformly considered for treatment with chemotherapy. However, response to treatment is markedly variable, and patients with TNBC still have higher rates of distant relapse than patients with any other BC subtype. Therefore, a greater understanding of the heterogeneity of these tumors and a more efficient classification system that highlights targetable differences is urgently needed to improve the treatment and outcome of patients with TNBC.

## The Origins of TNBC Subtyping

During the last decade, several groups invested their efforts into characterizing TNBC at different molecular levels. The first attempts to stratify TNBC were based on histology, immunohistochemistry (IHC), and transcriptomic profiling (**Table 1**). In 2013, TNBC was classified using 13 IHC markers by Elsawaf et al. This study identified four groups according to the expression patterns of cytokeratins (CK). After subsetting, patients with luminal (20%) or basoluminal (28%) displayed a significantly worse survival than patients with basal A (26%) or basal B (26%) TNBC tumors (11).

**Table 1:** Examples of TNBC stratification methods.

Classification Method	Subtypes	Freq (%)	Effect on prognosis	Characteristics
Histochemistry (11)	Luminal	20	Worse	EGFR<10%, Ki-67<50%, 2 or more luminal CK+
	Basoluminal	28	Worse	EGFR>10%
	Basal A	26	Better	EGFR<10%; high proliferation (Ki-67>50%)
	Basal B	26	Better	EGFR<10%, Ki-67<50%, 2 or luminal CK-
Gene expression from microarray (12)	BL1	18-26	Neutral	Cell cycle, DNA damage
	BL2	10-15	Neutral	Growth factor signaling
	IM	10-20	Neutral	Immune-related pathways
	M	12-20	Worse	Mesenchymal differentiation and proliferation
	MSL	8-16	Better	Mesenchymal features, low proliferation
	LAR	10-15	Worse	Hormone-related pathways, inflammation
Gene expression from microarray (13)	BL1	35	Better	Cell cycle, DNA damage
	BL2	22	Neutral	Growth factor signaling
	M	25	Neutral	Mesenchymal differentiation and proliferation
	LAR	16	Neutral	Hormone-related pathways, inflammation
Gene expression and CNV (14)	BLIA	49	Better	High proliferation, immune activation
	BLIS	23	Worse	High proliferation, immune suppression
	LAR	15	Neutral	Hormone-related pathways, inflammation
	MES	13	Neutral	Mesenchymal differentiation and proliferation
Gene expression (15)	C1	23	Better	Apocrine
	C2	41	Neutral	Basal-like, Immune suppression
	C3	36	Neutral	Basal-like, Immune checkpoint upregulation
mRNA and lncRNA expression (16)	MES	34	Neutral	EMT, lower levels of proliferation
	BLIS	32	Worse	Proliferative pathways, immunosuppression
	LAR	17	Neutral	Hormone-related pathways, inflammation
	IM	17	Neutral	Immune signaling
Alternative Polyadenylation (17)	LAR	22	Neutral	Hormone-related pathways
	MLIA	22	Neutral	Mesenchymal and Immune-related pathways
	BL	40	Neutral	DNA-damage response
	S	16	Worse	Cell growth, immune-related pathways
DNA methylation, 450K (18)	Epi-CL-A	25	Neutral	Mesenchymal differentiation and proliferation
	Epi-CL-B	33	Worse	DNA-damage response Cell division
	Epi-CL-C	22	Neutral	Hypoxia, protein degradation
	Epi-CL-D	20	Neutral	Immune-related pathways
DNA methylationMBDCap-Seq (19)	Cluster 1	58	Better	Largely hypomethylated
	Cluster 2	18	Neutral	High methylated
	Cluster 3	24	Worse	Medium methylated
Protein levels (20)	I/H-subtype	66	Neutral	Hormone-related pathways, inflammation
	DD-related	34	Neutral	DNA-damage response
Metabolic pathways (21)	MPS1	26	Neutral	Lipogenic
	MPS2	37	Worse	Glycolytic
	MPS3	37	Neutral	Mixed phenotype

### The Legacy of BC Subtyping Using Gene Expression Patterns

In 2011, Lehmann et al. identified six TNBC subtypes (TNBCtype-6 classification) based on gene expression profiling and ontology analyses (12). The novel subtypes included basal-like (BL) 1 and BL2, which were enriched in cell cycle genes and growth factor signaling, respectively; Immunomodulatory (IM), with high expression of immune-related pathways; mesenchymal (M), which presented genes of mesenchymal differentiation and proliferation; mesenchymal stem-like (MSL), which had mesenchymal features and low proliferation; and luminal androgen receptor (LAR), characterized by the activation of hormone-related pathways. Importantly, LAR and M subtypes had a significantly lower relapse-free survival than the rest of the subtypes. Five years later, the same group refined the classification since they observed an important presence of tumor-infiltrating lymphocytes (TILs) and stromal cells in the IM and MSL subtypes, respectively. Thus, the TNBC subtypes were refined as BL1, BL2, M, and LAR (TNBCtype-4 classification). BL1 displayed the best prognosis among the four subtypes (13). Similarly, Burstein et al. subdivided TNBC tumors using gene expression profiling and copy number variations (CNVs). They identified four stable groups with distinct prognoses and suggested putative subtype-specific targets. These subtypes were named LAR, mesenchymal (MES), basal-like immune-suppressed (BLIS), and basal-like immune-activated (BLIA). BLIS showed the worst survival, and BLIA the best survival compared to the rest (14). A recent study by Jézéquel et al. also employing transcriptomic profiling identified three different TNBC subtypes (C1, C2, and C3), taking advantage of the fuzzy clustering strategy. The C1 cluster included TNBC tumors with a molecular apocrine phenotype that showed a better prognosis, and C2 and C3 were enriched in basal-like properties. C2 displayed biological aggressiveness and an immune-suppressive phenotype, whereas C3 outlined the adaptive immune response and immune checkpoint upregulation (15).

Long-non-coding RNAs (lncRNAs) were considered to classify TNBC tumors by Liu et al., given their role as regulators of gene expression. They combined mRNA and lncRNA expression profiles (16) to construct the Fudan University classification (FUSCC) system. Similar to the findings by Lehman et al. and Burstein et al., four subtypes were identified: IM, enriched in immune cell signaling pathways; LAR, enriched in hormone-related pathways; MES, whose main features were low levels of cell proliferation-related genes and enriched pathways associated with epithelial-mesenchymal transition (EMT); and BLIS, showing upregulation of proliferative pathways and the downregulation of genes involved in the immune response. Again, patients with BLIS TNBC showed a worse overall prognosis. The authors compared these clusters with the TNBCtype-6 classification. They found that the IM groups were nearly identical in both studies, and LAR and BLIS partially covered Lehmann’s LAR and BL1 groups, respectively. The authors also designed an IHC-based approach to classify TNBC patients (22). Quist et al. employed a four-gene signature to cluster TNBC tumors into six subtypes. The MC6 subtype, which comprised nearly 50% of TNBC samples, was associated with a higher sensitivity to platinum-based chemotherapy. Importantly, this correlation was further validated in TNBC cell lines (23).

### From DNA to Metabolites for TNBC Clustering

New ways of stratifying TNBC patients have arisen thanks to the advent of next-generation sequencing, computing systems, and the exponential increase of available data sources during the following years. Thereby, new data types have been used to classify TNBC into novel subtypes (**Table 1**). Different single nucleotide variant (SNV) patterns have been identified in TNBC tumors (24) and circulating DNA from TNBC patients (25). Jiang et al. explored these differences in the FUSCC cohort. They discovered that somatic mutations and CNVs events were not homogeneously distributed among TNBC subsets. For instance, FUSCC LAR tumors were enriched in PI3K pathway mutations. High genomic instability was associated with the FUSCC BLIS subtype. Given the mutational differences, this study defined four genetic subtypes: Homologous recombination deficiency (HRD), clock-like, APOBEC, and mixed (26). Interestingly, the HDR subtype showed a greater proportion of germline variants than other mutation subtypes. *BRCA1*, *RAD51D*, and *BRCA2* were the most frequently mutated genes (27).

The mRNA processing machinery has also been considered to establish TNBC subtypes with analysis of alternative polyadenylation events in a TNBC cohort using a Bayes-based strategy. The gene expression of these four subtypes was then compared with TNBCtype-6 subtypes. Subtype 1, named LAR, was enriched in hormone-regulated pathways and displayed a significant overlap with the TNBCtype-6 LAR subtype. Taking this classification as a reference, gene expression patterns found in M, MSL, and IM groups were found in subtype 2, termed mesenchymal-like immune activated (MLIA). Subtype 3 was called basal-like (BL) due to increased proliferation and DNA damage-related genes, similar to the TNBCtype-6 BL subtype. Subtype 4, which exerted the worst prognosis, showed downregulation of cell growth and immune-related pathways and was identified as the Suppressed (S) subtype (17). Alternative splicing was also used to stratify TNBC tumors and identify a model to predict the overall survival. Alternative promoter signature significantly separated TNBC patients into high- and low-risk groups, suggesting that it might play a special role in the development and progression of TNBC (28).

DNA methylation (DNAm) patterns are also being implemented for tumor characterization (29, 30). Initially, three different TNBC DNAm clusters were identified using MBDCap-Seq. This study identified 865 TNBC differentially-methylated regions (DMR), most of them hypermethylated. Survival analysis showed that the TNBC subgroup, which included hypomethylated tumors, displayed a better prognosis (19). Most recently, we tried to explain the TNBC heterogeneity using DNAm profiles. Four TNBC epitypes were identified using machine learning (18). The Epi-CL-A was mainly composed of samples identified as mesenchymal according to the TNBCtype-6 classification. Epi-CL-B, which presented a worse prognosis, partially overlapped with the TNBCtype-6 LAR and the Burstein et al. BLIS subtypes. Consistent with the overlap mentioned above, Epi-CL-A showed activation of mesenchymal cell differentiation and proliferation pathways. In contrast, most of the differentially expressed genes in Epi-CL-B were involved in DNA damage response, maintaining chromatin structure, and cell division. Epi-CL-C was characterized by the activation of hypoxia-related pathways and the modulation of protein homeostasis. Finally, Epi-CL-D showed enrichment in immune-related pathways such as response to interferon-beta, positive regulation of T cell-mediated cytotoxicity, or antigen processing and presentation (18).

The tumor proteome analysis is another feature that is starting to see precision medicine applications as an approach for patient stratification. For example, two stable clusters of TNBC tumors were identified by reverse-phase protein array (RPPA). Analysis of the protein signatures revealed that one of the clusters was enriched in growth arrest and DNA damage (GADD45 genes) and p53 signaling pathways. This subtype was identified as the DNA damage (DD)-related subtype. Another cluster was designated as I/H-subtype due to its association with inflammation, hormonal receptor, and MAPK signaling pathways (20). Deeper subsetting defined the existence of 5 RPPA subtypes. Researchers found significant agreement between the RPPA classification system and the TNBCtype-4 system (13) and intrinsic subtypes by PAM50. Thus, the TNBCtype-4 subtypes BL1 and BL2 were enriched in the RPPA subclusters 2 and 1, respectively, classified as DD-related. The RPPA cluster 4, a subset of I/H-subtype, was enriched in PAM50 normal-like TNBC tumors (20). Additionally, integrative analysis of the proteome and genome identified potential protein markers of drug sensitivity and drug resistance. For example, enrichment with mitochondrial proteins was associated with sensitivity to drugs that might depend on mitochondrial protein expression, like belinostat (31). Similarly, a comprehensive quantitative proteome profile of BC cell lines identified two major subgroups within TNBC cell lines (basal A and B) with different functional signatures (32).

Most recently, metabolic pathways were also exploited as a differential feature to classify TNBC. Gene expression from the FUSCC cohort (26) was analyzed to identify transcriptional differences in genes involved in metabolic pathways (21). Thus, TNBC tumors were classified into three different molecular pathway subtypes (MPS) based on the enrichment scores of metabolic pathways. MPS1 was defined as the lipogenic subtype; MPS2 was characterized as a glycolytic subtype and showed the worst relapse-free survival among the three metabolic subtypes. Tumors that showed a mixed enrichment were identified as MPS3 subtype. Untargeted metabolomic analysis on frozen TNBC samples revealed that MPS1 presented higher amounts of fatty acids, whereas MPS2 showed higher levels of glycolysis mediators. Furthermore, they found that cell lines classified as MPS1 showed a higher fatty acid uptake and a higher sensitivity against C75, a *de novo* lipid synthesis inhibitor. In contraposition, glycolysis inhibitors displayed a more powerful growth inhibitory effect in those cells stratified as MPS2. Interestingly, based on this classification, the researchers were able to sensitize MPS2 against PD-1-targeted therapy through an LDH inhibitor, which decreased lactate levels, promoting immune recognition (21).

### The Microenvironment, a Novel Source of Information and Noise for Subtype Discovery

TNBC tumors have also been stratified according to their immunogenomic profile. The analysis of 29 immune-associated gene sets defined three clusters in four BC datasets. The subsets were called immunity low, immunity medium, and immunity high. The latter group was characterized by greater immune cell infiltration and anti-tumor immune activities associated with a better prognosis (33). The tumor microenvironment is also known to impact TNBC outcome, defining response subtypes. TNBC presents the highest proportion of TILs in comparison with other BC subtypes (34). Its presence is associated with a better prognosis (35, 36), higher rates of complete pathological response (pCR) to neoadjuvant chemotherapy, and better response to immunotherapy (37, 38). In another study, a high number of TILs was associated with enhanced survival. In contrast, increased levels of the immunosuppressor markers, such as PD-L1, CD163, and FOXP3, or a glycolytic microenvironment, determined by MCT4 expression, predicted a worse outcome. Together, these parameters were used to subset 174 TNBC tumors into four clusters. Clusters 1 and 2 defined by high TILs and low PD-L1 and FOXP3 showed better survival than clusters 3 and 4, both associated with increased PD-L1, FOXP3, and stromal MCT4 (39).

## Current and Future Applications

Precision treatment of TNBC is not utopic, and it is only just knocking on the door. Characterization of driver alterations in potentially druggable genes is essential to assess TNBC heterogeneity and tailor the best treatment for each patient (40). Molecular stratification and differential treatment assignment have been used in patients with refractory metastatic TNBC (ClinicalTrials.gov identifier: NCT03805399). The study revealed clinical benefits in IM and BLIS+BRCA1/2 wild-type subsets. Patients whose malignancies were classified as IM received a combination of paclitaxel + anti-PD-1, which promoted an objective response rate (ORR) in 50% of patients (41). Previous trials using monotherapy in TNBC without stratification showed an ORR of around 5-10% (42, 43). These preliminary data suggest that the overly simplistic current IHC classification of TNBC alone betrays the diverse heterogeneity of this subgroup and risks leaving on the table potential treatment options that can be effective if directed toward specific intrinsic subtypes.

In early-stage TNBC, TILs evaluation has been standardized (44) and should be routinely performed due to its prognostic value (45). In advanced TNBC, germline BRCA1/2 mutations are associated with higher response to platinum compounds (46). These mutations are routinely assessed to identify candidates for PARP inhibitor therapy (47, 48). Ongoing studies evaluate the incorporation of PARP inhibitors in other germline mutations beyond BRCA1/2, like PALB2, or in somatic BRCA1/2 mutations. PD-L1 expression by IHC is assessed to select patients for immunotherapy associated with chemotherapy (49, 50). Patients with metastatic TNBC harboring PIK3CA/AKT1/PTEN alterations have longer progression-free survival when treated with AKT inhibitors and chemotherapy (51, 52). Identifying tumor-associated antigens overexpressed in a subpopulation of TNBC may prompt the generation of new therapeutic strategies (53).

For TNBC that are classified into the immune-activated subtype, new biomarkers are emerging to predict response to immunotherapy in addition to PD-L1, such as the presence of TILs (54), tumor mutation burden (55), expression of immune genes (56), or through the construction of personalized cancer immunograms that integrate multiple variables (57).

### What Is Clear About the Still Fuzzy TNBC Subtyping?

**Figure 1:**
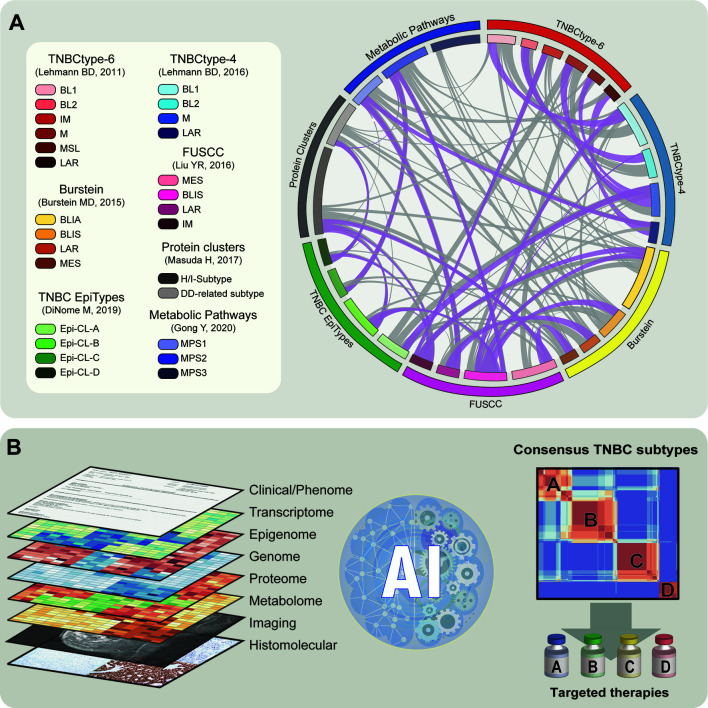
Illustrative representation of current subtypes and the future of subsetting in TNBC. **(A)** Left panel: Summary of TNBC classification methods described and their subtypes. Right panel: Representation of the similitude between the different classification systems that reported comparisons with existing methods. Ribbons represent the partial overlap between different subtypes. Ribbons referring to strong overlaps are shown in purple. **(B)** Left panel: Schematic representation of the different layers of information to construct the definitive TNBC subtypes. This includes clinical, molecular, and histological data. Middle plot: Representation of application of artificial intelligence (AI) algorithm to integrate diverse datasets and construct TNBC subtypes. Right panel: Schematic correlation plot representing consensus integrative TNBC subtypes. TNBC stratification can be applied to improve subtype-specific therapies.

Perhaps, the several strategies employed to construct systems that identify clinically useful TNBC subtypes reflect the lack of proper definition of this disease. From the quantitative variables such as gene and protein expression or metabolic and epigenomic profiling to the qualitative traits such as gene mutation, basic and translational researchers have explored a problem that still appears to remain unsolved. We see a common factor, independently of the approach, that clear overlaps exist between the different classification systems (**Figure 1A**). This is encouraging as it points towards the existence of stable entities identified in diverse patient populations. However, the fact that there is still a large variability, added to several subtle similitudes between some of the current subtyping systems (grey ribbons **Figure 1A**),** **suggests that there is still a long way to go.

### In Pursuit of the Consensus TNBC Subtypes

In the upcoming years, integrating different data sources will be key in identifying definitive TNBC subtypes that will help guide clinicians toward specific treatment recommendations for their patients. Integrative analyses comparing TNBC and non-TNBC patients from the TCGA cohort have been performed combining gene expression, DNAm, and somatic mutations, revealing differential signatures between these two types of BC (58). Thus, a similar approach combining even more layers of information may identify consensus TNBC subtypes. There is already one attempt to stratify TNBC patients using multiple data types, specifically using transcriptome (RNA-seq), micro-RNA expression (miRNA-seq), and CNV (59). However, this study did not use metabolomics, proteomics, imaging, or histomolecular features, which have independently proven to be informative for subtyping TNBC.

### Beyond the Human’s Good Intentions, Is Artificial Intelligence the Key?

The efforts of several scientists led to the generation of a substantial amount of knowledge about TNBC heterogeneity, which is intended to improve precision treatments. Nevertheless, there is still a wealth of static and dynamic data due to clinical parameters and treatment perturbations that escape from the analytical skills employed to construct the subtyping systems. It seems clear that the key to constructing a definitive and clinically useful classification of TNBC subtypes will incorporate integration of all the datasets and subtyping systems created to date. We believe that recent advances in artificial intelligence (AI) will accelerate this process and provide the largely anticipated rational stratification system for TNBC patients (**Figure 1B**).

Most of the current subtyping systems have relied on information from a single data source. However, the complexities of TNBC biology are unlikely captured sufficiently by a single data type. Instead, the combined information across multiple data types can provide a more holistic view of the complexities of TNBC biology. Advancements in the AI subfields of machine learning and deep learning have produced powerful methods that can be leveraged to construct models using diverse molecular data types (60, 61). The power of these methods lies in the ability to capture more complex relationships within data than traditional statistical approaches. Thus, these methods provide the necessary tools to integrate the diverse molecular data of current TNBC subtype systems. Furthermore, deep learning methods effectively extract information from non-molecular data types such as clinical imaging and histopathology (62), significantly improving the current stratification methods. As the speed and scale at which biological data is collected increases and new advancements in computational technology emerge, AI-based methods will increasingly provide a powerful analytical framework for analyzing molecular and clinical data. Without question, these parallel advancements will constitute a breakthrough in TNBC precision diagnosis and treatment, addressing the most aggressive form of BC.

## Author Contributions

All authors contributed to the article and approved the submitted version.

## Funding

This study was supported by the Instituto de la Salud Carlos III Miguel Servet Project (#CP17/00188) and AES2019 (#I19/01514), the Institut d’Investigació Sanitària Illes Balears (IdISBa) FUTURMed FOLIUM program, the Associates for Breast and Prostate Cancer Studies (ABCs) Foundation, the Fashion Footwear Association of New York (FFANY) Foundation, the Asociación Española Contra el Cancer (AECC) Foundation, the Balearic Islands Government Margalida Comas program, the Fundación Francisco Cobos, and the UCLA Breast Cancer Epigenetics Research Program.

## Conflict of Interest

The authors declare that the research was conducted in the absence of any commercial or financial relationships that could be construed as a potential conflict of interest.
